# Simultaneous utilization of CO_2_ and potassium-rich biomass for the environmentally friendly production of potassium formate†

**DOI:** 10.1039/d4ra07360f

**Published:** 2025-01-03

**Authors:** Hayoung Yoon, Kwangho Park, Kwang-Deog Jung, Sungho Yoon

**Affiliations:** a Department of Chemistry, Chung-Ang University 84 Heukseok-ro, Dongjak-gu Seoul Republic of Korea sunghoyoon@cau.ac.kr; b Clean Energy Research Center, Korea Institute of Science and Technology (KIST) 5 Hwarang-ro 14-gil, Seongbuk-gu Seoul 02792 Republic of Korea jkdcat@kist.re.kr

## Abstract

The C_1_ chemical species, potassium formate (K(HCO_2_)), known as a two-electron reducing agent, finds application in the synthesis of multi-carbon compounds, including oxalate, and plays a crucial role not only in the food and pharmaceutical industries but also across various sectors. However, the direct hydrogenation of CO_2_ to produce K(HCO_2_) remains a challenge. Addressing this issue, efficient production of K(HCO_2_) is achieved by integrating CO_2_ hydrogenation in a trickle-bed reactor using a heterogeneous catalyst with a novel separation method that utilizes potassium ions from biomass ash for formic acid derivative product isolation. Through alkaline-mediated CO_2_ hydrogenation using *N*-methylpyrrolidine (NMPI), a concentrated 5 M NMPI solution of formic acid *N*-methylpyrrolidine complex ([NMPIH][HCO_2_]) was formed, facilitating the synthesis of K(HCO_2_) with over 99% purity *via* reaction with excess K ions contained within Bamboo ash. Notably, 80% of CO_2_ was converted to formate ions, and NMPI was expected to be effectively recycled as it was completely removed during the evaporation process for K(HCO_2_) separation. Additionally, this process yielded SiO_2_ by-product particles with sizes ranging from 10 to 20 nm. This research highlights a novel strategy contributing to sustainable environmental management and resource recycling by effectively utilizing CO_2_ as a valuable feedstock while concurrently producing valuable chemical compounds from waste materials.

## Abstract 2

K(HCO_2_) and SiO_2_ were co-produced using an environmentally friendly method that simultaneously utilized formate derivatives generated through CO_2_ hydrogenation and bamboo ash, a K-rich biomass.

## Graphical abstract

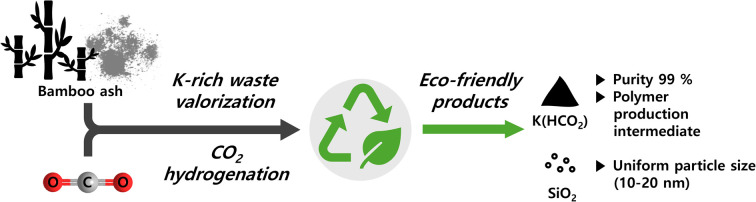

## 1. Introduction

The increase in atmospheric CO_2_ concentration is widely recognized as a primary driver of recent climate change.^1,2^ To mitigate further increases and even reduce current levels of atmospheric CO_2_, extensive research has been directed towards various strategies.^1,2^ Among these, the chemical utilization of CO_2_ to produce valuable chemicals, known as Carbon Capture and Utilization (CCU), is particularly intriguing.^1,2^ This approach, often termed CO_2_ valorization, primarily yields chemical species such as carbonates, CO, formic acid, methanol, methane (single-carbon compounds), and oxalic acid, acetic acid, ethylene glycol, and ethylene (two-carbon compounds).^1–15^ Among these, formic acid is notable due to its production *via* the reaction of CO_2_ and H_2_, achieving 100% atom economy without by-products.^12^ This makes formic acid a distinctly advantageous ‘green’ product compared to other CO_2_ conversion products.

Recently, numerous studies have reported on catalysts and processes for the conversion of CO_2_ to formic acid, with some technologies pilot-scales of up to 10 kg day^−1^.^16^ However, despite these advancements, there are significant limitations to the application of CO_2_-to-formic acid technology as a climate change mitigation strategy, particularly in terms of societal acceptability.^12,17,18^ Currently, formic acid is used primarily as a reducing agent in chemical reactions, in leather tanning, and as a preservative in animal feeds,^19–22^ with the reported global market demand in 2023 being approximately 920 000 tons per year.^23^ This market size inherently limits the potential of CO_2_-to-formic acid technology to significantly reduce atmospheric CO_2_, given the vast amount of CO_2_ that needs to be mitigated globally.

To overcome this limitation, one promising approach is to develop technologies that also produce formate species, which are extensively used in current industrial processes.^24–28^ This strategy could potentially enhance the overall impact of CO_2_ conversion technologies by expanding the range of valuable products that can be derived from CO_2_. Recent research has focused on enhancing the diversity of CO_2_ conversion products by developing methods for producing formate-based compounds such as metal formates.^24–28^ Metal formates, depending on the type of metal ion, can be converted into various chemical species including formaldehyde, oxalate, and methyl formate, thus possessing high market potential.^5,27–34^ Therefore, the study of various methods for producing metal formates is expected to significantly contribute to mitigating global warming by advancing applicable CO_2_ conversion technologies in society.

**Fig. 1:**
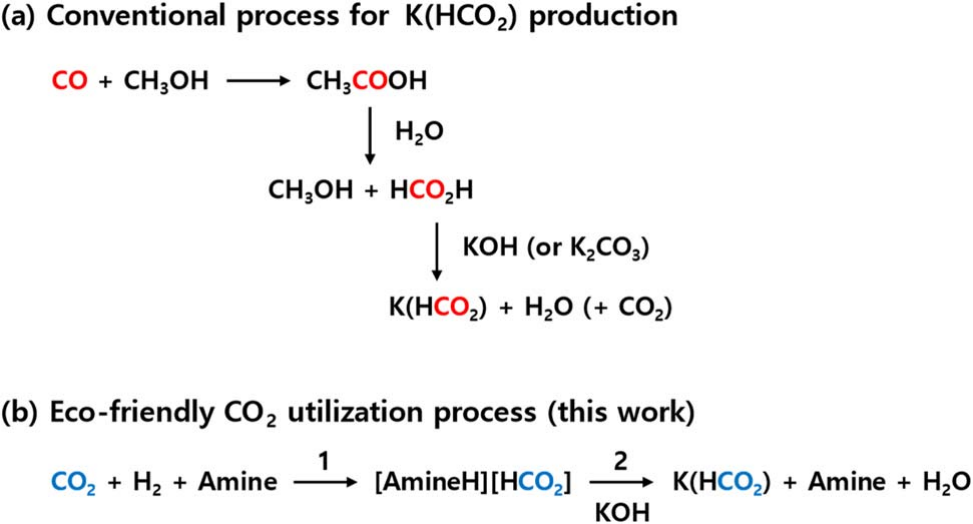
K(HCO_2_) production routes: (a) conventional process and (b) eco-friendly CO_2_ utilization process.

A representative example of such research has demonstrated the production of calcium formate using CO_2_ hydrogenation products and various calcium ion sources.^25,26^ This study suggested the possibility of generating other metal formates from CO_2_ hydrogenation products, though research extending beyond calcium ions to other metal formates has not yet been reported. Interestingly, recent advancements have proposed a novel approach using potassium formate (K(HCO_2_)) to synthesize the C_2_ compound oxalate, further advancing the production of multi-carbon chemical species such as C_3_ and C_4_ compounds.^27,28^ If K(HCO_2_) can be efficiently produced from CO_2_, it holds significant potential as an intermediate for generating oxalate from CO_2_. However, research in this area remains scant. The currently known methods for producing K(HCO_2_) involve adding KOH or K_2_CO_3_ to formic acid (Fig. 1a).^35^ This approach, however, entails high production costs of formic acid and issues related to CO_2_ emissions during the production process of formic acid from CO.^36,37^ Therefore, to facilitate the production of various useful carbon resources from CO_2_, there is a critical need for the development of efficient CO_2_ conversion technologies for producing K(HCO_2_).

The utilization of readily accessible resources for the production of CO_2_ conversion products is crucial for ensuring cost-effective resource supply and enhancing the practical application of these processes from research to commercialization in society.^25,26,38^ Among these approaches, the method of producing K(HCO_2_) from waste-derived potassium oxide contributes significantly to sustainable environmental management and resource circulation. Biomass ash, rich in potassium oxide, can be effectively used in such research. Biomass itself holds substantial potential as an energy source, and consequently, the quantity of resulting biomass ash is expected to increase.^39^ In this context, employing potassium oxide from biomass ash represents an effective strategy to simultaneously achieve two critical objectives: CO_2_ conversion and waste valorization.

Building on this background, a method was developed for synthesizing K(HCO_2_) from formic acid derivatives through CO_2_ hydrogenation in an amine-based alkaline aqueous solution (eqn (1)), followed by using potassium ions contained in biomass ash to separate the formic acid derivatives (eqn (2)). Using amine as a base, this single-step CO_2_ hydrogenation process achieved a 72% reduction of CO_2_, producing a 5 M amine high concentration of [AmineH][HCO_2_]. This was then reacted with K ions from bamboo ash, resulting in K(HCO_2_) with a purity exceeding 99% and, additionally, producing silica (SiO_2_) nanoparticles with particle sizes of 10–20 nm as a by-product. This method benefits from bypassing the complex processes of producing K(HCO_2_) *via* formic acid from CO_2_, allowing for direct production of K(HCO_2_) from CO_2_ hydrogenation products (Fig. 1b). This approach offers an innovative method for extracting K ions from various wastes, including biomass ash, contributing to resource circulation and CO_2_ reduction.

Hydrogenation:

1 Amine + CO_2_ + H_2_ → [AmineH][HCO_2_]

Separation:

2 [AmineH][HCO_2_] + KOH → K(HCO_2_) + Amine + H_2_O

## 2. Experimental

### 2.1. Materials and instrumentation

All reagents, except for bamboo charcoal (BC) and the catalyst, were purchased from Sigma-Aldrich and were used without further purification. The reagents used are as follows: CaO (99.9% trace metals basis), MgO (97%), KOH (anhydrous, ≥99.95%), SiO_2_ (nano powder, 10–20 nm particle size (BET), 99.5% trace metals basis), NMPI (97%) and formic acid (≥95%). BC was sourced from Suncheon, South Korea. The catalyst Ru/bpyTN-CTF used for CO_2_ hydrogenation was used the same as that in the existing formic acid production reaction.^16,40^

The concentration of the formic acid salt was measured using a Waters Alliance 2695 system that was equipped with a refractive index detector and an Aminex HPX-87H high-performance liquid chromatography (HPLC) column. All the HPLC analysis results were obtained at a flow rate of 0.6 mL min^−1^ and a column temperature of 50 °C in a 5.00 mM H_2_SO_4_ mobile phase. ^1^H-NMR and ^13^C-NMR spectra were recorded on 600 MHz ^1^H (150 MHz ^13^C) spectrometer (VNS, Varian) using D_2_O and CDCl_3_ as solvents. Fourier transform infrared (FT-IR) spectroscopy analysis was conducted using the pellet method and a Nicolet iS10 instrument with a mercury cadmium telluride detector (Thermo Fisher Scientific). X-ray diffraction (XRD) analysis was performed using a New D8-Advance diffractometer (Bruker-AXS), and X-ray fluorescence (XRF) spectroscopy was performed using a ZSX Primus II system (Rigaku). Inductively coupled plasma optical emission spectroscopy (ICP-OES) was applied to the determination of the elements K, Ca, Mg, and Si using an Agilent ICP-OES 720 system (Agilent). Field-emission scanning electron microscopy (FE-SEM) and energy-dispersive X-ray spectroscopy (EDS) were performed at 20.0 kV accelerating voltage using a Sigma 300 instrument (Carl Zeiss). Thermogravimetric analysis (TGA) was performed using SDT Q600 (TA Instruments Ltd), under a N_2_ atmosphere (flow of 20 mL min^−1^) from 24 °C to 1000 °C at a heating rate (5 °C min^−1^).

### 2.2. Preparation of degassed-[NMPIH][HCO_2_] (2)

#### 2.2.1. Hydrogenation of CO_2_ in a trickle-bed reactor to produce [NMPIH][HCO_2_] (1)^16^

The CO_2_ hydrogenation reaction was conducted in a trickle-bed reactor. A total of 1.0 g of Ru/bpyTN-CTF powder containing 3 wt% Ru was loaded in the center of the vertically aligned reactor. Prior to initiating the reaction, the reactor was pressurized to 12 MPa under varying feed gas (CO_2_ : H_2_ = 1 : 1.5) flows and heated to 120 °C. Additionally, H_2_O and NMPI were supplied at 3.54 and 0.68 mol h^−1^, respectively. Gas flow rate was controlled by mass flow controller, and liquid flow rate was controlled by high-pressure liquid pump. The experimental conditions included a space velocity was set at 24 cm s^−1^.

Following the reaction, liquid product 1 was obtained and analyzed using HPLC and NMR spectroscopy analyses to confirm the concentration of formate and the acid to amine ratio (AAR). The conversion achieved was 72%, the AAR was measured at 0.72, the productivity was 356.7 g_form._ g_cat._^−1^ day^−1^, and the turnover frequency (TOF) was 103 h^−1^.

#### 2.2.2. Thermal degassing of 1 for preparation of 2

Transparent liquid 2 was prepared by heating 1, produced through CO_2_ hydrogenation, at 90 °C for 2 h. The composition of 2 was confirmed using ^13^C-NMR spectroscopy, ^1^H-NMR spectroscopy, and HPLC analyses.

### 2.3. Production of metal formates using metal oxides and 2

Details of this process are provided in Section 1 of the ESI.†

### 2.4. Production of K(HCO_2_) using bamboo ash and 2

#### 2.4.1. Preparation of 800 °C calcined bamboo ash (800dBA)

800dBA was prepared by calcining BC at 800 °C for 24 hours to use it in a form suitable for producing K(HCO_2_). As result of calcining 2 g, the weight was reduced by a total of 27.4%. After calcining under these conditions, the black BC turned gray, and the resulting calcined bamboo ash was then subjected to both qualitative and quantitative analyses through XRF and ICP-OES measurements. Additionally, the completion of the calcination process was confirmed using TGA.

#### 2.4.2. Extraction of K compound from bamboo ash

1 g of 800dBA, prepared by calcination at 800 °C for 24 h, was added to 10 mL of water and stirred at 100 °C for 12 h to 7 days to extract K compounds. Quantitative analysis of the extracted metal elements was performed using ICP-OES.

#### 2.4.3. Production of K(HCO_2_) using K compound extracted from bamboo ash and 2

1.0 mL of 2 (HCO_2_^−^: 5.31 mmol) was added into a 50 mL 2-neck round bottom flask, followed by the addition of 25.6 mL of solution (K: 0.8 wt%) containing K compound (K: 5.31 mmol) extracted from 800dBA. The mixture was stirred at 30 °C for 5 min, then the reaction solution was evaporated at 90 °C under 10^−3^ mbar. The resulting solid was redissolved in water and the precipitated white SiO_2_ solid was separated by filtration. The filtrate was then evaporated at 80 °C under 10^−3^ mbar to yield a white solid, K(HCO_2_). Quantitative analysis of the produced K(HCO_2_) was conducted using HPLC, and its purity was confirmed *via* ICP-OES analysis. (Yield: 99.8%) The morphology of the separated SiO_2_ was analyzed using FE-SEM, and XRD was performed to further confirm its crystalline structure. Additionally, XRD analysis was carried out on the produced K(HCO_2_) to verify its identity.

## 3. Result & discussion

### 3.1. Optimizing amine selection for enhanced CO_2_ hydrogenation in K(HCO_2_) production

In 2022, a method was developed to produce calcium formate (Ca(HCO_2_)_2_) from formic acid triethylamine complex aqueous solution ([*x*Et_3_NH][*y*HCO_2_], where *x* and *y* represent the molar ratios of triethylammonium (Et_3_NH^+^) and formate (HCO_2_^−^) ions, respectively), a product of CO_2_ hydrogenation in an aqueous triethylamine solution (eqn (3)).^25^ Initially, the composition of [1.0Et_3_NH][0.74HCO_2_] following CO_2_ hydrogenation was 2.77 M Et_3_N and 2.03 M HCO_2_H. However, considering that the solubility of Ca(HCO_2_)_2_, is only 16.6 g per 100 mL of water,^41^ the amount of solvent initially present was insufficient for the formation of Ca(HCO_2_)_2_. To allow for the formation of the desired amount of Ca(HCO_2_)_2_, additional water had to be supplied. Consequently, the solution was diluted to reduce the HCO_2_H concentration to 1.71 M. This dilution led to significant energy consumption during the subsequent evaporation phase needed to isolate the final product.

3 2[Et_3_NH][HCO_2_] + CaO → Ca(HCO_2_)_2_ + 2Et_3_N + H_2_O

In contrast, the solubility of K(HCO_2_) is considerably higher at 331 g per 100 mL,^42^ eliminating the need for any dilution. Utilizing higher concentrations of formate adduct for the production of K(HCO_2_) can thus enhance process efficiency by minimizing the quantity of water required.

Furthermore, recent research has utilized *N*-methylpyrrolidine (NMPI) as a base for CO_2_ hydrogenation, producing formic acid *N*-methylpyrrolidine complex aqueous solution ([*x*NMPIH][*y*HCO_2_], where *x* and *y* represent the molar ratios of *N*-methylpyroolidinium (NMPIH^+^) and formate (HCO_2_^−^) ions, respectively).^16^ This process achieved a composition of 5.05 M NMPI and 3.64 M HCO_2_H, representing a reduction in water usage by approximately 37% compared to when 2.77 M Et_3_N is used to produce 2.03 M HCO_2_H (Table S5†). Based on these results, it has been decided to use [1.0NMPIH][0.72HCO_2_] (1) for the production of K(HCO_2_), leveraging the higher solubility and reduced water requirement of this approach.

### 3.2. Production of high-concentration [NMPIH][HCO_2_] through CO_2_ hydrogenation

[1.0NMPIH][0.72HCO_2_] (1), utilized for the production of K(HCO_2_), was synthesized *via* CO_2_ hydrogenation under conditions of 120 °C and 120 bar (H_2_/CO_2_ = 1.5) in a trickle-bed reactor (TBR) using a Ru/bpyTN-CTF catalyst (Fig. S1†).^16^ In one reaction cycle, 80% of CO_2_ is converted to formate ions. The composition of the produced 1 was confirmed to be 5.05 M NMPI and 3.64 M HCO_2_H, with the presence of bicarbonate also detected by ^13^C-NMR spectra (eqn (4) and Fig. S2(a)†). The presence of bicarbonate can potentially reduce the yield of metal formates by reacting with metal ions to form metal carbonates instead of metal formates. Therefore, a degassing process was considered to remove bicarbonate before using the solution for the production of K(HCO_2_) (eqn (5)).

4 NMPI + CO_2_ + H_2_ → 0.72[NMPIH][HCO_2_] + 0.28[NMPIH][HCO_3_]

5 0.72[NMPIH][HCO_2_] + 0.28[NMPIH][HCO_3_] → 0.72[NMPIH][HCO_2_] + 0.28NMPI + 0.28H_2_O + 0.28CO_2_↑

This degassing process was conducted to prepare [1.0NMPIH][1.07HCO_2_] (2) at 90 °C for 2 h, and the removal of bicarbonate was verified by ^13^C-NMR spectroscopy analysis. Furthermore, the composition of 2 was analyzed using ^1^H-NMR spectroscopy and HPLC. ^13^C-NMR spectra showed that the bicarbonate peak observed at 160.92 ppm in the pre-degassed sample was completely eliminated after degassing (Fig. S2(b)†). Compositional changes before and after degassing were confirmed through ^1^H-NMR spectroscopy and HPLC analyses. ^1^H-NMR spectra indicated a shift in the ratio of NMPI to HCO_2_H from 1 : 0.72 before degassing to 1 : 1.07 afterward (Fig. S3†), resulting from partial evaporation of NMPI and H_2_O. The evaporated NMPI and H_2_O can be captured and recycled back into the CO_2_ hydrogenation process, preventing any loss of NMPI. Additionally, HPLC analysis calculated the composition of 2 as 4.96 M NMPI and 5.31 M HCO_2_H, demonstrating that the amount of HCO_2_H remained stable through the degassing process without decomposing or evaporating. Consequently, 2 was applied in the production of K(HCO_2_).

### 3.3. Selection and characterization of K-rich biomass ash for K(HCO_2_) production

To identify a suitable reactant for the production of K(HCO_2_), various types of biomass ash were examined for their high potassium content. Biomass ash generally contains major elements such as Ca, Mg, K, and P, which vary depending on the type of biomass. Therefore, selecting the appropriate biomass based on the desired metal content is crucial when utilizing biomass ash. For instance, Clean wood pellet ash is rich in Ca and Mg, while agro-residue ashes such as maize stalk ash, maize cob and cotton stalk ash predominantly contain K and P.^43–45^ In this study, the composition of various agro-residue ashes was analyzed to select a biomass ash rich in K (Table S6†). Bamboo ash was found to contain 53.4 wt% K, significantly higher than that found in other biomass-derived ashes. The notably high-potassium content in bamboo ash can be attributed to the rapid growth of bamboo, which necessitates abundant mobile potassium, an essential nutrient for plant growth.^45,46^ Consequently, this high-potassium content renders bamboo ash an effective reactant for the production of K(HCO_2_).

The supplied BC was calcined at 800 °C for 24 h for use in the K(HCO_2_) production reaction. This temperature was selected based on TGA (Fig. S4†), which indicated no further weight loss beyond 800 °C, confirming complete decomposition. This ensured that the ash was fully processed and suitable for analysis and reaction. 800dBA was then subjected to XRF qualitative analysis to identify its elemental composition (Table 1). The results showed that the main metal elements in 800dBA were K, Si, Ca, and Mg, with relatively higher proportions of K and Si compared to Ca and Mg. These findings align with the previously studied inorganic elemental composition of bamboo ash (Table S6†), where K accounted for nearly 50 wt%, significantly higher than the other metal elements. Subsequent quantitative analysis of these metal elements in 800dBA was performed using ICP-OES (Table S7†). The ICP-OES analysis revealed that K constituted the highest proportion at 63.4 mol%, followed by Si at 14.1 mol%, Ca at 12.4 mol%, and Mg at 10.4 mol%. This confirms the predominant presence of K, further demonstrating its suitability for K(HCO_2_) production.

**Table 1:** Relative elemental composition except light elements of 800dBA for 24 h measured by XRF analysis

Component		K	Si	Ca	P	Mg	S	Al	Fe	Mn	Ba	Zn	Cl	Ti	Na
Result	wt%	43.5	34.9	8.8	6.3	2.1	1.7	0.8	0.8	0.5	0.2	0.1	0.1	0.1	0.1

**Fig. 2:**
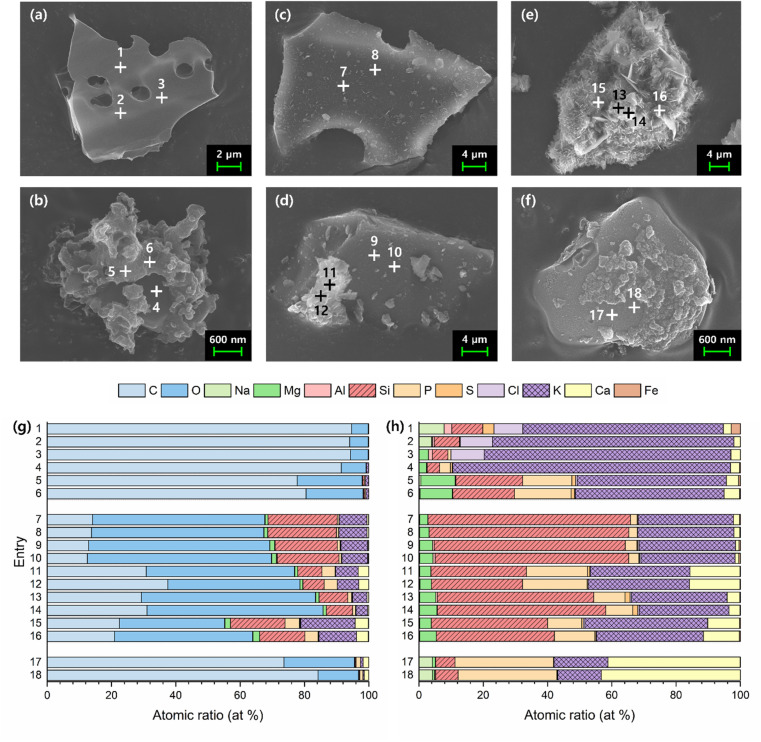
FE-SEM images (a–f) and EDS point analysis graphs (g and h) of BC ((a and b), entry 1–6), 800dBA ((c–e), entry 7–16), and solid obtained by filtration after stirring 800dBA in water at 100 °C for 5 days ((f), entry 17, 18).

FE-SEM and EDS analyses were conducted to examine the morphology and corresponding elemental composition trends of BC and 800dBA (Fig. 2 and Table S8†). BC exhibited a mixture of particles with irregular morphologies of varying sizes, which could be broadly classified into two types (Fig. 2a and b). The first type of particle (a) displayed a smooth surface with pore-like structures, while the second type of particle (b) exhibited an irregular, rough texture. In particular, the rough appearance of particle (b) seemed to cover a smooth surface, which appeared similar to that of particle (a). EDS point analysis was performed on three spots per particle, revealing that both types of particles contained a high proportion of C (Fig. 2g Entry 1–6), implying that BC sample did not achieve complete pyrolysis. In addition, spots 1–4 exhibited similar elemental compositions, where K was the most abundant element after C and O, alongside detectable amounts of Si and Cl. The rough surface of the particle (b) revealed higher proportions of Mg and P (Fig. 2h Entry 1–6). This indicates that BC likely contains phases such as KCl, K_2_SiO_3_, K–Mg silicates, phosphates, and sulfates.

In the case of 800dBA, it consisted of a mixture of particles with varying sizes and morphologies (Fig. 2c–e). The first type of particle (c) appeared flat but not smooth, while the second type of particle (e) contained a combination of two distinct crystalline forms. Spots 13 and 14 revealed thin, amorphous crystals layered over each other, whereas hexagonal crystals were observed at spots 15 and 16. The third type of particle (d) displayed a surface similar to that of particle (c), with the addition of crystals akin to those observed in particle (e). EDS point analysis of these particles showed a substantial decrease in C content in 800dBA compared to BC, accompanied by a significant increase in elements such as Si and K (Fig. 2g Entry 7–16). Furthermore, when excluding C and O, Si and K were found to be the dominant elements, with the proportion of Ca and P varying depending on the crystalline form (Fig. 2h Entry 7–16). These findings suggest that 800dBA contains K–Mg/Ca silicates, phosphates, and sulfates.

To investigate the mineral phase transformation behavior of BC after calcination at 800 °C, XRD analysis was performed (Fig. S5†). Prior to calcination, KCl and SiO_2_ were prominently identified, along with the presence of K_2_SO_4_ and KHCO_3_. However, following calcination, the peaks for KCl and KHCO_3_ disappeared, suggesting that KCl evaporated into the gas phase, and KHCO_3_ decomposed thermally, converting into other K-containing compounds such as K_2_SO_4_.^47^ Additionally, the emergence of KCaPO_4_ and Ca_15_(PO_4_)_2_(SiO_4_)_6_ peaks after calcination indicates that under the condition of 800 °C, sintering occurred between K and Ca with Si and P. It can also be inferred that K and Mg, which are prone to sintering with Si and P, likely contributed to the formation of other compounds such as K–Mg silicates, phosphates, and sulfates.^47^

### 3.4. Synthesis of metal formate using various metal oxide from degassed-[NMPIH][HCO_2_] (2)

**Fig. 3:**
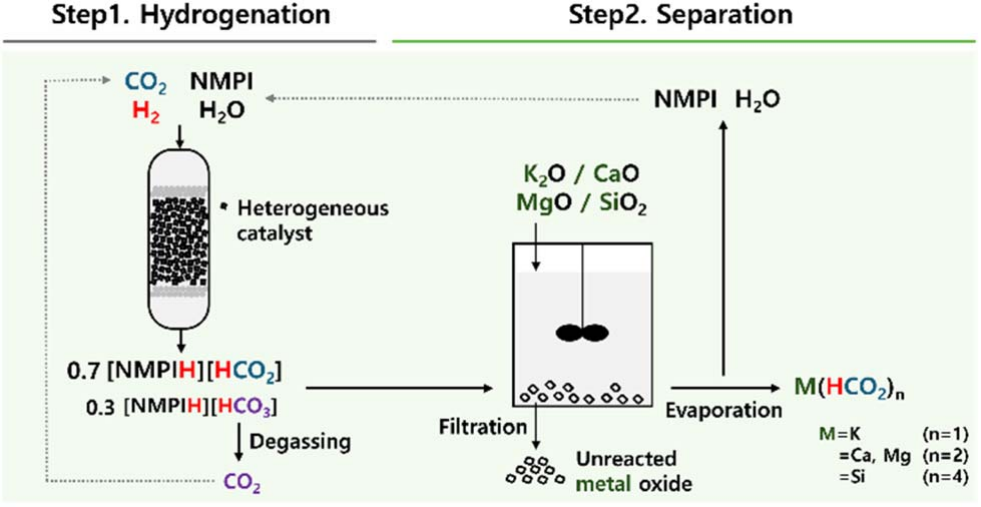
Schematics of the process for producing metal formate using various metal oxides from CO_2_ hydrogenation adduct made under *N*-methylpyrrolidine (NMPI)-based basic aqueous solution.

Prior to applying 800dBA directly for the production of K(HCO_2_), the oxides of four major metals constituting 800dBA were added to 2 for the synthesis of metal formates (Fig. 3). The process of producing metal formates from 2 involves adding metal oxides to 2, followed by stirring at specified temperatures and times. This process results in the formation of metal formates, while the unreacted metal oxides are separated through filtration. The filtrate contains the dissolved metal formates, and by evaporating this filtrate, NMPI and H_2_O are separated from the products, ultimately yielding solid metal formates. This method enables the efficient separation of high-purity metal formates.

Two key aspects were verified in synthesizing metal formates using this method. First, the complete separation of NMPI and H_2_O during the evaporation process of the filtrate was analyzed, followed by measuring the yield of the generated metal formates.

Since NMPI can be recycled to the CO_2_ hydrogenation process, completely separating it from the filtrate is crucial. The temperature at which NMPI and H_2_O evaporate from the filtrate was determined, and the separated liquids at each temperature were identified using ^1^H-NMR spectroscopy analysis. The known boiling point of NMPI is 80–81 °C,^48^ and for H_2_O, it is 100 °C. Therefore, evaporation was conducted at 90 °C to first separate NMPI. ^1^H-NMR spectroscopy analysis of the liquid separated at this temperature confirmed the presence of only NMPI (Fig. S6(a)†). It was found that only about 1.04% of the initially added NMPI remained in the remaining filtrate (Fig. S6(b)†). Subsequently, evaporation of H_2_O was performed at 90 °C, 10^−3^ mbar, and the remaining solid was analyzed using ^1^H-NMR spectroscopy, confirming that only K(HCO_2_) remained, with no NMPI present (Fig. S6(c)†).

To compare the reactivity of converting K, Si, Ca, and Mg oxides, each a major component of 800dBA, into their respective metal formates, equivalent amount of these oxides was added to 2 and stirred at 30 °C for 5 min. Subsequently, the yield of the produced metal formates was examined. Specifically, for K, KOH was used as a reactant instead of K_2_O, as potassium oxide is highly unstable under normal conditions and readily converts to KOH in the presence of moisture. The concentration of 2 was adjusted considering the solubility at 20 °C of the metal formates (Table 2). For K(HCO_2_), which has a solubility of 331 g/100 mL H_2_O, dilution was unnecessary, hence undiluted 2 was used. For Ca and Mg, considering the solubilities of 16.6 g/100 mL H_2_O for Ca(HCO_2_)_2_ and 14.4 g/100 mL H_2_O for Mg(HCO_2_)_2_,^49^ dilution processes were conducted. The resulting compositions for the diluted 2 used in the Ca(HCO_2_)_2_ and Mg(HCO_2_)_2_ experiments were set at 1.77 M NMPI and 1.90 M HCO_2_H, and 1.76 M NMPI and 1.88 M HCO_2_H, respectively. Si(HCO_2_)_4_ was used at the same concentration as the Ca experiment due to a lack of solubility information.

**Table 2:** Chemical reactions for metal formates production using various metal oxides with 2, compositions of 2 used, and yields of metal formates produced^a^

	Reaction equation^b^	NMPI [M]	HCO_2_H [M]	Metal formate yield [%]
K	2[NMPIH][HCO_2_] + K_2_O → 2K(HCO_2_) +2NMPI + H_2_O	4.96	5.31	99.0
Ca	2[NMPIH][HCO_2_] + CaO → Ca(HCO_2_)_2_ + 2NMPI + H_2_O	1.77	1.90	65.7
Mg	2[NMPIH][HCO_2_] + MgO → Mg(HCO_2_)_2_ + 2NMPI + H_2_O	1.76	1.88	20.4
Si	4[NMPIH][HCO_2_] + SiO_2_ → Si(HCO_2_)_4_ + 4NMPI + 2H_2_O	1.77	1.90	N/A

a The reactions were conducted at 30 °C for 5 min. The yields were calculated as an average from the results of two experiments.

b [NMPIH][HCO_2_] is 2.

The yields of each metal formate were as follows (Table 2). Given the hygroscopic properties of K(HCO_2_), its weight was measured inside a glove box to exclude the effects of moisture from the air. As a result, K(HCO_2_) was produced with a high yield of 99.0%, whereas Ca(HCO_2_)_2_, Mg(HCO_2_)_2_, and Si(HCO_2_)_4_ were generated with yields of 65.8%, 20.4%, and 0.1%, respectively. While all four metals are thermodynamically capable of converting to metal formate salts, the incomplete conversion of Ca, Mg, and Si can be inferred from factors such as kinetic limitations and the differences in the solubility of the resulting formate salts. These variations in yield can be attributed primarily to the difference in reactivity of the metal oxides with the formate ion, where basic oxides are more reactive than acidic oxides. Notably, alkali metal oxides like K_2_O exhibit higher reactivity compared to alkaline earth metal oxides such as CaO and MgO. Within the group of alkaline earth metals, CaO, owing to its higher solubility in hydroxide form, reacts more readily than MgO, which contributes to its relatively higher yield in the formate form. These intrinsic differences in chemical behavior among the metal oxides are crucial for the selective conversion of K compounds to K(HCO_2_) from bamboo ash. However, depending on reaction conditions, Ca and Mg may also be converted into metal formates. In particular, when Ca, which has higher reactivity after K, coexists with K, it was observed that the proportion of Ca converted to metal formate increases as the reaction time is extended and the temperature rises (details are provided in ESI 2†). Therefore, a pretreatment process to remove Ca and Mg from bamboo ash is essential to maintain the high purity of K(HCO_2_).

### 3.5. Synthesis of K(HCO_2_) using potassium compound extracted from bamboo ash and degassed-[NMPIH][HCO_2_] (2)

**Fig. 4:**
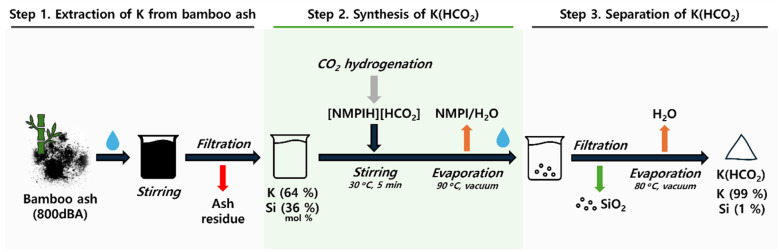
Production process of K(HCO_2_) from 800dBA and 2.

To effectively produce K(HCO_2_) using 800dBA, the potassium compound was initially extracted by stirring 800dBA in water, followed by reacting the extracted potassium solution with 2. This process comprises three stages (Fig. 4): first, the extraction of K from 800dBA; second, the addition of the extracted potassium solution to 2 to produce K(HCO_2_); and third, obtaining high-purity K(HCO_2_) as the final product. Through this method, K(HCO_2_) is expected to be produced with high yield and purity.

Optimization of the K extraction process from 800dBA was conducted by stirring at 100 °C over time. 1 g of 800dBA was added to 10 mL of water, and the extraction was set from 12 h up to 7 days. ICP-OES analysis (Fig. S7 and Table S9†) showed an increasing trend in K extraction with longer stirring times at 100 °C. However, after 5 days, the rate of increase in K extraction notably decreased, suggesting this point as the optimal extraction time. Interestingly, it was found that SiO_2_, generally insoluble in water, was also extracted. This indicates that the solution became sufficiently alkaline to dissolve SiO_2_ due to the presence of KOH extracted from bamboo ash. Additionally, Ca and Mg were found to be minimally extracted (Table S9†), leading to a solution containing 64 mol% K and 36 mol% Si obtained after stirring for 5 days at 100 °C. Furthermore, EDS analysis was conducted to identify the elements present in the solid residue after Si and K extraction (Fig. 2f–h Entry 17, 18). The results revealed a significant reduction in the Si and K content, while a substantial amount of C remained despite the sample being calcined at 800 °C.

The solution from which K was extracted was then added to 2 to produce K(HCO_2_) using the following procedure: the mixture was stirred at 30 °C for 5 min, then the reaction mixture was evaporated at 90 °C under vacuum to remove NMPI and H_2_O. The solid obtained from evaporating the filtrate was redissolved in water, insoluble solids were filtered out, and the remaining filtrate was evaporated at 80 °C under vacuum to yield a white solid. Redissolving the evaporated solid in water was aimed at precipitating and separating SiO_2_, which does not dissolve in K(HCO_2_) solution, thus efficiently producing high-purity K(HCO_2_).

**Fig. 5:**
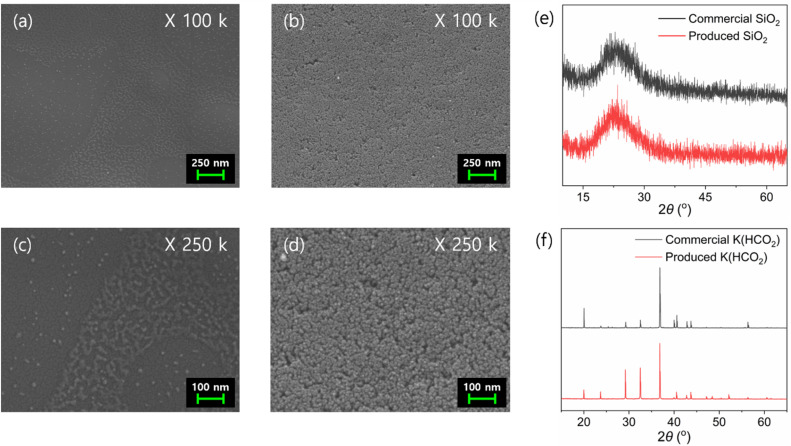
FE-SEM images of commercial SiO_2_ (a and c), SiO_2_ filtered during the K(HCO_2_) separation step (b and d), XRD spectrums of commercial SiO_2_ (black) and SiO_2_ filtered during the K(HCO_2_) separation step (red) (e), and XRD spectrums of commercial K(HCO_2_) (black) and K(HCO_2_) produced by using KOH and 2 (red) (f).

ICP-OES analysis of the precipitated solid from the redissolved solution confirmed that the material was 95.7% Si, identifying it as SiO_2_ (Table S10†). FE-SEM analysis confirmed that the particles ranged in size from 10–20 nm (Fig. 5a–d). Further XRD analysis verified the material as amorphous SiO_2_ (Fig. 5e). Finally, XRD analysis of the solid product obtained through evaporation confirmed that it had the same XRD pattern as commercial K(HCO_2_) (Fig. 5f). In addition, HPLC analysis showed that 99.8% of the initially added formate was contained within the solid (Table S11†), suggesting nearly complete conversion of the extracted K compound to K(HCO_2_). Additional ICP-OES analysis of the produced K(HCO_2_) confirmed a purity of 99.0% (Table S12†). These results demonstrate the successful production of K(HCO_2_) from a CO_2_ hydrogenation adduct using K-rich bamboo ash.

The process described above has successfully demonstrated the potential to produce K(HCO_2_) using formic acid derivative generated from CO_2_ hydrogenation and bamboo ash. This innovative approach validates the feasibility of synthesizing a valuable chemical from CO_2_ and sustainably sourced biomass ash, highlighting an effective utilization of CO_2_ and biomass waste.

## 4. Conclusion

This study has successfully demonstrated an innovative method for producing K(HCO_2_) from formic acid derivative produced through CO_2_ hydrogenation using K-rich biomass ash, specifically from bamboo. This method not only offers a practical way to utilize CO_2_ but also enhances the valorization of agricultural waste, contributing significantly to sustainable environmental management.

Utilizing NMPI as a base, the research achieved the hydrogenation of CO_2_ to produce a high concentration of the formic acid derivative [NMPIH][HCO_2_]. This compound subsequently reacted with potassium extracted from bamboo ash, yielding K(HCO_2_) with an exceptional purity of 99%. The method optimized the extraction of potassium from bamboo ash and showed that a prolonged extraction period at 100 °C efficiently obtains soluble potassium compounds, suitable for the synthesis of high-purity K(HCO_2_). Importantly, this process enabled the conversion of 80% of CO_2_ into K(HCO_2_) in a single cycle.

This findings provide a viable pathway for the production of valuable chemical compounds by effectively using greenhouse gases and biomass waste, highlighting the dual benefits of resource recovery and CO_2_ conversion. The approach is adaptable to various types of biomass and can be extended to synthesize different metal formates, providing a versatile platform for industrial applications. Furthermore, the successful production of K(HCO_2_) from CO_2_ paves the way for developing processes to create other multi-carbon chemicals, such as oxalic acid, further expanding the scope of sustainable chemical production methods.

## Data availability

The data used to support the findings of this study are included within the article.

## Author contributions

Hayoung Yoon: conceptualization, methodology, data curation, investigation, visualization, validation, writing – original draft, and writing – review and editing. Kwangho Park: data curation, investigation, visualization, validation, writing – original draft, and writing – review and editing. Kwang-Deog Jung: funding acquisition, project administration, resources, and writing – review and editing. Sungho Yoon: conceptualization, funding acquisition, project administration, resources, supervision, and writing – review and editing.

## Conflicts of interest

There are no conflicts of interest to declare.

## Supplementary Material

RA-015-D4RA07360F-s001
