# Supplementary material for: Simultaneous utilization of CO2 and potassium-rich biomass for the environmentally friendly production of potassium formate
Source: RSC Adv. 2025 Jan 3;15(1):348–56. doi: 10.1039/d4ra07360f (PMC11696524; doi:10.1039/d4ra07360f)
Supplement: RA-015-D4RA07360F-s001 [file RA-015-D4RA07360F-s001.pdf]

Supplementary Information (SI) for RSC Advances.
This journal is © The Royal Society of Chemistry 2025

Supplementary information

Simultaneous Utilization of CO_2 and Potassium-rich Biomass for the Environmentally Friendly Production of Potassium Formate

Hayoung Yoon, †[a] Kwangho Park, [b] Kwang-Deog Jung*[b] and Sungho Yoon*[a]

[a]Department of Chemistry, Chung-Ang University, 84 Heukseok-ro, Dongjak-gu, Seoul, Republic of Korea. E-mail: sunghoyoon@cau.ac.kr

[b]Clean Energy Research Center, Korea Institute of Science and Technology (KIST), 5 Hwarang-ro 14-gil, Seongbuk-gu, Seoul 02792, Republic of Korea. E-mail: jkdcat@kist.re.kr

Contents

1. Supplemental Experimental Procedures of production of metal formate using various metal oxides and bamboo ash directly

2. Results of experiments on direct use of bamboo ash for production of $K(HCO_2)$ (Table S1-S4)

- Figures S1 to S7

- Tables S5 to S12

1. Supplemental Experimental Procedures of production of metal formate using various metal oxides and bamboo ash directly

1.1. Production of $K(HCO_2)$ using K_2O

K_2O reacts rapidly with atmospheric moisture to form KOH, making it unsuitable for experimental use. Therefore, KOH was employed instead. In the glove box, KOH (106 mmol, 5.95 g) was placed into a 50 mL 2-neck round bottom flask (RB) and securely attached to a condenser. The flask was then moved outside the glove box, and 20 mL of degassed-[NMPIH][HCO_2] **(2)** ([1.0NMPIH][1.07HCO_2]) was added. The mixture was stirred at 30 °C for 5 min. To remove *N*-metylpyrrolidine (NMPI), the mixture was evaporated at 90 °C under atmospheric pressure. Subsequent vacuum evaporation was performed at 90 °C and 10^{-3} mbar to remove all liquids, resulting in a white solid. The solid was weighed inside the glove box, and its yield of $K(HCO_2)$ was determined using HPLC analysis (Yield: 99.0 %)

1.2. Production of $Ca(HCO_2)_2$ using CaO

In a 50 mL 2-neck RB, 20 mL of **2** was introduced, followed by the addition of water (14.8 g) to adjust the concentration of $NMPIH^+$ to 1.77 M and HCO_2^- to 1.90 M. Subsequently, CaO (19.0 mmol, 1.06 g) was added. The mixture was stirred at 30 °C for 5 min. To remove NMPI, evaporation was conducted at 90 °C under atmospheric pressure. Following this, vacuum evaporation at 90 °C and 10^{-3} mbar was performed to remove all liquids, resulting in a white solid. The solid was then dried under vacuum at 120 °C. (Yield: 65.8 %)

1.3. Production of $Mg(HCO_2)_2$ using MgO

In a 50 mL 2-neck RB, 20 mL of **2** was introduced, followed by the addition of water (14.8 g) to adjust the concentration of $NMPIH^+$ to 1.75 M and HCO_2^- to 1.88 M. Subsequently, MgO (18.8 mmol, 0.758 g) was added. The mixture was stirred at 30 °C for 5 min. To remove NMPI, evaporation was conducted at 90 °C under atmospheric pressure. Following this, vacuum evaporation at 90 °C and 10^{-3} mbar was performed to remove all liquids, resulting in a white solid. The solid was then dried under vacuum at 120 °C. (Yield: 20.4 %)

1.4. Production of $Si(HCO_2)_4$ using SiO_2

In a 50 mL 2-neck RB, 20 mL of **2** was introduced, followed by the addition of water (14.8 g) to adjust the concentration of $NMPIH^+$ to 1.77 M and HCO_2^- to 1.90 M. Subsequently, SiO_2 (4.75 mmol, 0.285 g) was added. The mixture was stirred at 30 °C for 5 min. To remove NMPI, evaporation was conducted at 90 °C under atmospheric pressure. Following this, vacuum evaporation at 90 °C and 10^{-3} mbar was performed to remove all liquids, resulting in a white solid. The solid was then dried under vacuum at 120 °C. (Yield: 0.1 %)

1.5. Production of $K(HCO_2)$ using bamboo ash directly

In a 50 mL 2-neck RB, 1.0 mL of **2** (formate 5.31 mmol) was added, followed by the addition of water to bring the total volume to 20 mL. Subsequently, 1.8216 g of 800 °C calcined bamboo ash (800dBA) containing 11.4 wt% K (K:5.31 mmol) was added. The mixture was stirred at 30 °C for 2 h, after which by-products were removed through filtration. The filtrate was then subjected to vacuum evaporation at 90 °C and 10^{-3} mbar to obtain a white solid. Quantitative analysis of the $K(HCO_2)$

contained in the white solid was conducted using HPLC, and the purity of the produced $K(HCO_2)$ was verified through ICP-OES analysis.

2. Results of experiments on direct use of bamboo ash for production of $K(HCO_2)$

To assess the conversion rates to formate metal salts of the most reactive components, KOH and CaO, found in 800dBA, reaction conditions were varied with temperatures set at 30 °C and 100 °C, and reaction times at 5 min and 120 min (Table S1). In entries 1-3, 0.5 eq. of KOH were added, while in entries 4 and 5, 1.0 eq. of KOH was added. The results indicated that the addition of 0.5 eq. each of KOH and CaO consistently produced nearly 100 % $K(HCO_2)$ irrespective of reaction time and temperature. However, as reaction time and temperature increased, the yield of $Ca(HCO_2)_2$ also increased. Conversely, when 1.0 eq. of KOH and 0.5 eq. of CaO were used, $K(HCO_2)$ was still produced nearly completely, but $Ca(HCO_2)_2$ was hardly formed, even as reaction time progressed. These outcomes suggest that when using 800dBA mixed with various metal oxides, if K compounds are completely converted to $K(HCO_2)$, the formation of other metal formates is not expected even with increased reaction time. However, if $K(HCO_2)$ is not fully formed, an increase in reaction time and temperature may lead to the formation of Ca and Mg formates, potentially decreasing the purity of $K(HCO_2)$.

Table S1. Yields of metal formates produced using mixed KOH and CaO with **2**.[a]

Entry	Temp. [°C]	Time [min]	NMPI [M]	HCO_2H [M]	K^+ [eq.]	Ca^{2+} [eq.]	$K(HCO_2)$ Yield [%]	$Ca(HCO_2)_2$ Yield [%]
1	30	5	4.96	5.31	0.5	0.5	100	0.08
2	30	120	4.96	5.31	0.5	0.5	100	17.7
3	100	5	4.96	5.31	0.5	0.5	100	56.5
4	30	5	4.96	5.31	1.0	0.5	96.0	0.48
5	30	120	4.96	5.31	1.0	0.5	96.7	0.29

[a] 0.5 eq. of metal ion: 2.65 M, 1.0 eq.: 5.31 M. The yield of $Ca(HCO_2)_2$ was calculated assuming that the yield of $K(HCO_2)$ in entries 1-3 was 100 %, and the yields of $K(HCO_2)$ and $Ca(HCO_2)_2$ in entries 4 and 5 were confirmed through ICP-OES analysis (Table S3).

The yield of $K(HCO_2)$ was determined by directly adding 800dBA to **2** and conducting the reaction at 30 °C for 2 h (Table S2). The yield of $K(HCO_2)$ was found to be 56.7 %, and its purity was 97.8 % as confirmed by ICP-OES analysis (Table S4). These results suggest that an increase in $K(HCO_2)$ production from 800dBA may require improved K extraction through a longer reaction time or a higher reaction temperature. However, these conditions may lead to the undesirable conversion of CaO to $Ca(HCO_2)_2$. Consequently, to achieve high yields of high-purity $K(HCO_2)$, it is essential to maximize K extraction by stirring 800dBA in water.

Table S2. Yields of metal formates produced using 800dBA with diluted-**2**.

Temp. [°C]	Time [min]	NMPI [M]	HCO_2H [M]	K^+ [M]	Ca^{2+} [M]	$K(HCO_2)$ Yield [%]	$Ca(HCO_2)_2$ Yield [%]
30	120	0.316	0.339	0.339	0.0662	56.7	0.08

* Main metal composition of 800dBA: K (63.4 mol%), Ca (12.4 mol%) (Table S6), and the yields of $K(HCO_2)$ and $Ca(HCO_2)_2$ were confirmed through ICP-OES analysis (Table S4).

Table S3. Metal composition of products resulting from the reaction of **2** with a mixture of KOH and CaO calculated by ICP-OES analysis.

**Table S1, Entry 4** Component	mg/kg	wt%	mol%
K	356212	99.4	99.5
Ca	2002	0.56	0.55

**Table S1, Entry 5** Component	mg/kg	wt%	mol%
K	350068	99.7	99.7
Ca	915	0.26	0.25

Table S4. ICP-OES analysis of evaporated solid produced using 800dBA (directly used) and **2**.

Component	wt%	mol%
K	98.5	97.8
Si	0.7	1.0
Ca	0.14	0.1
Mg	0.7	1.1

Table S5. Amounts of reactants and products in the CO_2 hydrogenation adducts when using Et_3N and NMPI.[1]

Amine	T	P	H_2O	amine	H_2	CO_2	[AmineH][HCO_2]	[AmineH][HCO_3]	AAR
	[^oC]	[bar]		[mol h^{-1}]			[M]	[M]	
Et_3N	120	120	3.66	0.34	0.68	0.68	2.03	0.73	0.74
NMPI	120	120	3.54	0.68	1.02	0.68	3.63	1.41	0.72

① The composition of [Et_3NH][HCO_2]

	[M]	[mmol]	[mL]
Et_3N	2.77	55.3	7.7
HCO_2H	2.03	40.7	1.5
H_2O		595	10.8
(Total V)			20.0

② The composition of [NMPIH][HCO_2]

	[M]	[mmol]	[mL]
NMPI	5.05	101	10.5
HCO_2H	3.64	72.8	2.7
H_2O		374	6.8
(Total V)			20.0

Figure S1. Schematic of CO_2 hydrogenation process through a trickle-bed reactor packed with Ru molecular catalyst, heterogenized on a bipyridine (bpy)-terephthalonitrile (TN) mixed-CTF (Ru/bpyTN-30-CTF).[1]

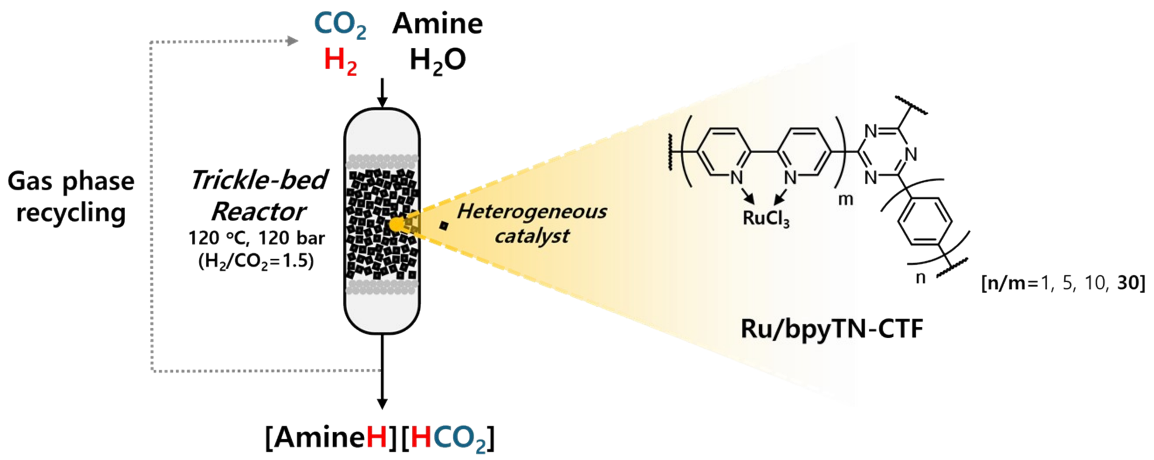

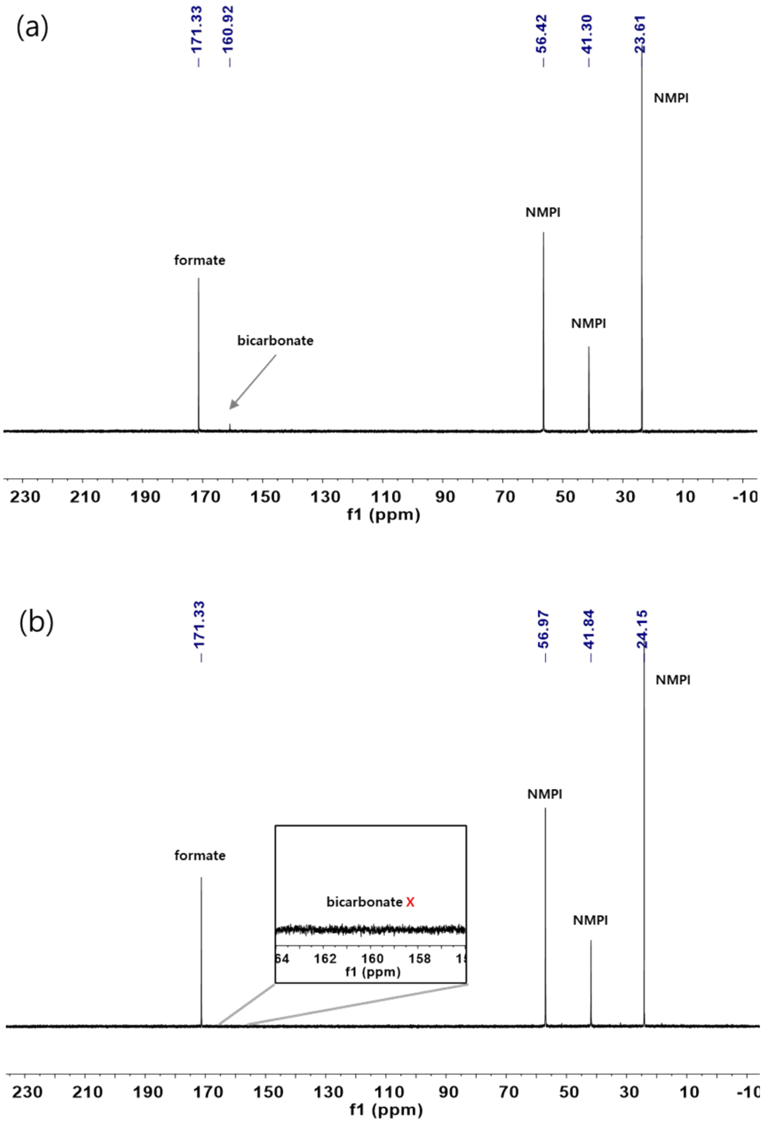

Figure S2. ^{13}C-NMR analyses of [NMPIH][HCO_2] adduct before (a) and after (b) degassing.

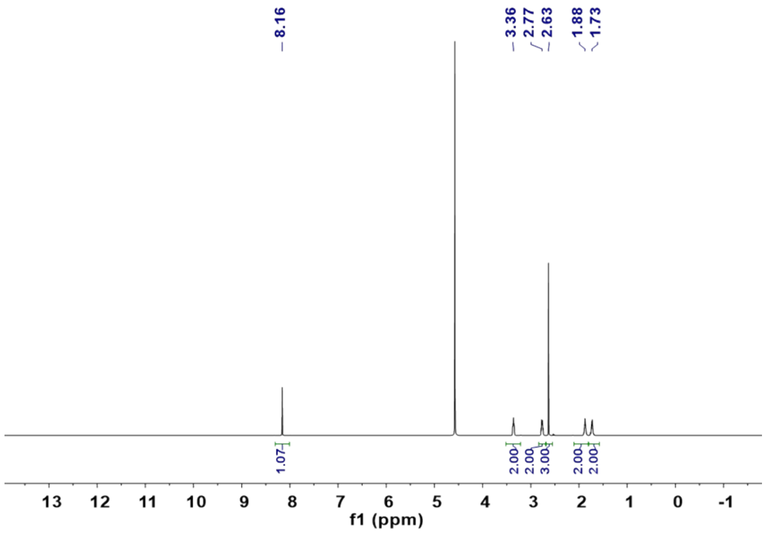

Figure S3. ^{1}H-NMR analysis of [NMPIH][HCO_2] adduct after degassing (**2**).

Table S6. Inorganic elemental composition of agro-residue ash.

Content (wt%)	Bamboo whole[2]	Miscan-thus[2]	Sorghum grass[2]	Sweet sorghum[2]	Switch-grass[2]	Wheat straw[2]	Rice husks[2]	Sugarcane bagasse[2]	Sunflower husks[2]	Wheat straw[3]	Soybean stalk[3]	Cotton stalk[4]	Maize stalk[5]	Maize cob[5]
Ti	0.01	0.03	0.02	0.06	0.28	0.09	0.02	2.02	0.15	-	-	0.1	-	-
S	3.68	2.28	1.11	3.47	0.83	4.24	0.92	3.57	4.07	-	-	5.69	1	1
Si	9.92	56.42	73.21	66.85	66.25	50.35	94.48	46.79	23.66	58	33.18	3.98	21	18
P	20.33	5.54	4.43	3.47	3.92	3.54	0.54	3.87	7.13	2.8	2.43	7.03	8	4
Na	0.31	0.47	0.25	1.47	0.58	3.52	0.16	1.61	0.8	0.9	3.56	7.81	<1	/
Mg	6.57	3.01	2.21	3.12	4.71	2.74	0.19	4.56	7.33	1.4	8.23	8.1	4	2
K	53.38	19.75	8.97	4.49	9.64	24.89	2.29	6.95	28.53	14	15.72	39.55	30	32
Fe	0.67	0.94	0.95	0.58	1.36	0.88	0.22	11.12	4.27	1.5	1.75	1.17	<1	<1
Cl	-	-	-	-	-	-	-	-	-	-	-	4.73	2	3
Ca	4.46	10.77	7.02	10.41	10.21	8.21	0.97	4.91	15.31	6	30.95	20.32	3	1
Al	0.67	0.79	1.83	0.81	2.22	1.54	0.21	14.6	8.75	3.2	2.55	1.52	1	1

Table S7. ICP-OES analysis of 800dBA.

Component	wt%	mol%
K	68.5	63.4
Si	14.2	14.1
Ca	15.0	12.4
Mg	7.0	10.4

Table S8. Chemical compositions of the Spots presented in Figure 2 (g, h), analyzed by EDS.

	at%											
Spot	C	O	Na	Mg	Al	Si	P	S	Cl	K	Ca	Fe
1	94.67	5.14	0.01	0	0	0.02	0	0.01	0.02	0.12	0	0.01
2	94.05	5.74	0.01	0	0	0.02	0	0	0.02	0.16	0	0
3	94.3	5.47	0	0.01	0	0.01	0	0	0.03	0.18	0	0
4	91.47	7.72	0	0.02	0	0.03	0.03	0	0	0.72	0.02	0
5	77.76	20.12	0.01	0.25	0	0.46	0.29	0.02	0.01	1	0.07	0.01
6	80.52	17.7	0.01	0.2	0	0.36	0.29	0.02	0	0.84	0.07	0
7	14.12	53.52	0.08	0.96	0	21.49	0.54	0.06	0.01	8.62	0.51	0.09
8	13.76	53.6	0.08	1.12	0	21.37	0.7	0.02	0	8.78	0.5	0.06
9	12.88	56.28	0.07	1.49	0.17	19.34	0.89	0.08	0.12	8.28	0.29	0.12
10	12.46	57.27	0.08	1.49	0.23	19.18	0.77	0.04	0.11	7.93	0.28	0.15
11	30.78	46.12	0.07	0.92	0.02	7.5	4.11	0.14	0.03	7.03	3.25	0.03
12	37.5	41.04	0.08	0.87	0.01	6.63	4.05	0.05	0	6.67	3.06	0.04
13	29.26	54.16	0.05	1.03	0.1	8.81	1.28	0.21	0.06	4.47	0.54	0.03
14	31.03	54.72	0.03	0.97	0.03	8.14	0.9	0.17	0.05	3.54	0.38	0.02
15	22.42	32.69	0.12	1.73	0.04	16.94	4.48	0.2	0.21	16.93	4.12	0.12
16	20.96	42.89	0.11	2.08	0.02	14.06	4.2	0.11	0.07	11.71	3.68	0.13
17	73.63	21.9	0.19	0.04	0	0.28	1.39	0.01	0	0.78	1.77	0
18	84.2	12.6	0.13	0.02	0.01	0.23	0.99	0	0.01	0.45	1.36	0

	at%									
Spot	Na	Mg	Al	Si	P	S	Cl	K	Ca	Fe
1	7.84	0	2.4	9.62	0	3.47	8.99	62.36	2.43	2.89
2	3.94	0.25	0.58	7.86	0	0.21	10.05	75.07	2.04	0
3	0	2.93	1.23	4.83	0.94	0	10.35	76.78	2.94	0
4	0	2.34	0.21	3.86	3.37	0.56	0.13	86.47	2.77	0.29
5	0.6	10.71	0.15	20.77	15.33	1.16	0.48	46.46	3.78	0.55
6	0.34	10.03	0.15	19.18	17.65	1.09	0.19	46.33	4.82	0.22
7	0.21	2.54	0	63.07	2.09	0.22	0.04	29.66	1.89	0.28
8	0.19	2.94	0	62.18	2.67	0.08	0.02	29.89	1.84	0.19
9	0.18	4.07	0.48	59.45	3.63	0.31	0.46	29.88	1.11	0.42
10	0.21	4.13	0.68	60.21	3.22	0.18	0.43	29.31	1.09	0.53
11	0.24	3.43	0.07	29.67	19.02	0.7	0.15	31.03	15.55	0.14
12	0.32	3.53	0.05	28.31	20.15	0.28	0	31.51	15.69	0.16
13	0.24	4.96	0.53	48.67	9.7	1.61	0.43	29.75	3.95	0.18
14	0.16	5.39	0.2	52.38	8.37	1.52	0.46	28.01	3.33	0.17
15	0.24	3.57	0.08	36.14	10.58	0.48	0.48	38.27	9.88	0.27
16	0.25	5.13	0.04	36.71	12.57	0.33	0.21	33.24	11.16	0.35
17	4.14	0.98	0.05	6.06	30.58	0.17	0.01	16.78	41.16	0.05
18	4.17	0.64	0.34	7.02	30.77	0.11	0.19	13.48	43.28	0

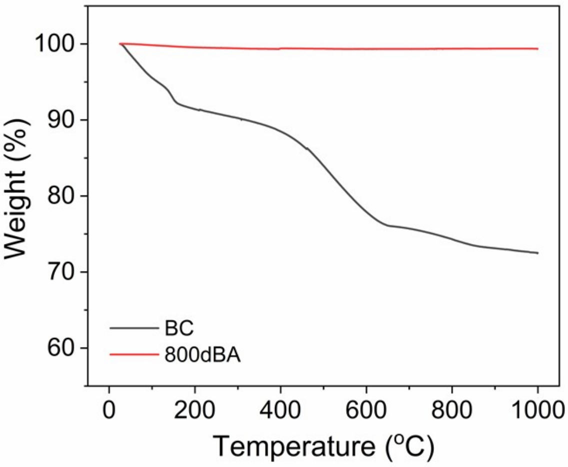

Figure S4. TGA plots of bamboo charcoal (BC) (black) and 800dBA (red).

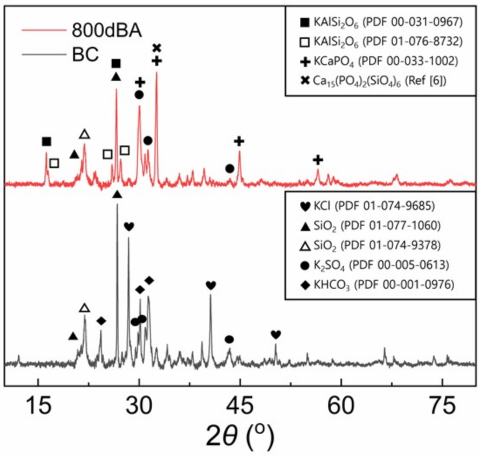

Figure S5. XRD spectrums of BC (black) and 800dBA (red).[6]

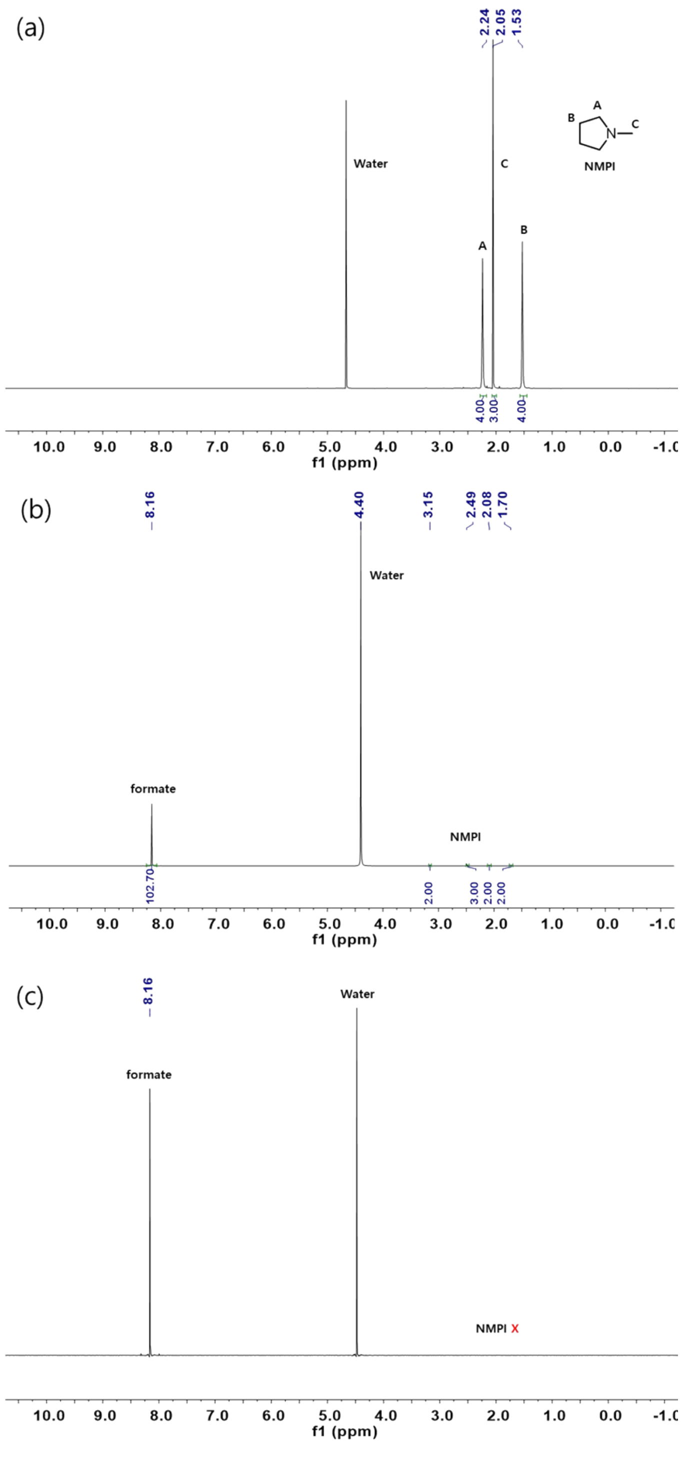

Figure S6. ^{1}H-NMR analyses of the liquid separated at 90 °C from the reaction mixture generated using KOH and **2** (a), the liquid remaining after separation at 90 °C (b), and solid obtained after evaporation of all liquid at 90 °C under 10^{-3} mbar (c).

Table S9. Metal concentrations in filtrate from 800dBA stirred in water over various time periods determined by ICP-OES[a].

Extraction time	12 h	1 d	3 d	5 d	7 d
component	[mg/kg]	[mg/kg]	[mg/kg]	[mg/kg]	[mg/kg]
K	2961	3628	5189	8119	8656
Si	1629	1649	3207	6661	7256
Ca	3.031	1.463	0.84	0.695	1.243
Mg	4.233	2.251	0.73	0.181	0.245

[a]0.5 g of 800dBA was added to 5 mL water and stirred for each hour at 100 °C.

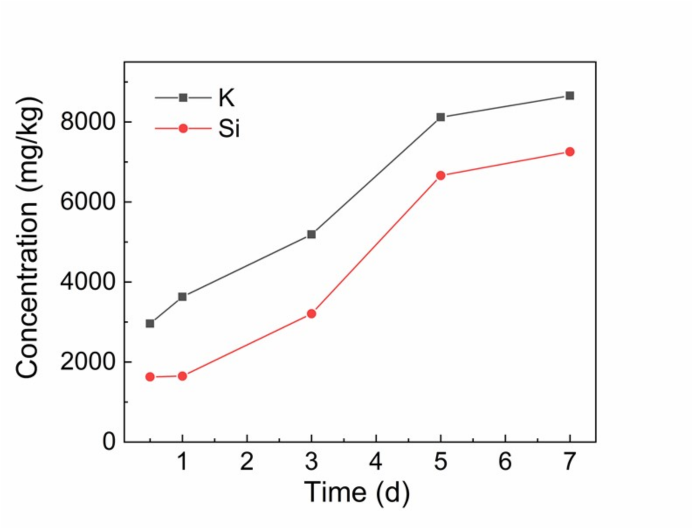

Figure S7. K and Si concentrations in filtrate obtained from 800dBA stirred in water over various time periods as determined by ICP-OES measurements.

Table S10. Metal composition of SiO_2 filtered during $K(HCO_2)$ separation step calculated by ICP-OES analysis.

Component	mg/kg	wt%	mol%
K	7964	5.76	4.21
Si	130211	94.2	95.7
Ca	87	0.06	0.04
Mg	24	0.02	0.02

Table S11. HPLC analysis results of 1 M HCO_2H and $K(HCO_2)$ produced using the solution containing K extracted from 800dBA and **2**.

	HPLC area	Expected concentration [M]	Measured concentration [M]	$K(HCO_2)$ Yield [%]
1M HCO_2H	13828874	-	-	-
Produced $K(HCO_2)$	1379757	0.1	0.0998	99.8

Table S12. ICP-OES analysis result of $K(HCO_2)$ solution produced using the solution containing K extracted from 800dBA and **2**.

Component	mg/kg	wt%	mol%
K	8525	99.3	99.0
Si	57.05	0.66	0.92
Ca	5.202	0.06	0.06
Mg	0.815	0.01	0.02

<Reference>

1. C. Kim, Park, K., Lee, H., Im, J., Usosky, D., Tak, K., ... & Lee, U, *Joule*, 2024, **8**, 693-713.
2. Q. Kang, L. Appels, T. Tan and R. Dewil, *The Scientific World Journal*, 2014, **2014**.
3. M. Variny, A. Varga, M. Rimár, J. Janošovský, J. Kizek, L. Lukáč, G. Jablonský and O. Mierka, *Processes*, 2021, **9**, 100.
4. H. Fan, F. Li, Q. Guo and M. Guo, *Journal of the Energy Institute*, 2020, **93**, 1781-1789.
5. A. Peys, H. Rahier and Y. Pontikes, *Applied Clay Science*, 2016, **119**, 401-409.
6. Y. Zhu, J. Hu, W. Yang, W. Zhang, K. Zeng, H. Yang, S. Du and H. Chen, *Energy & Fuels*, 2018, **32**, 5244-5251.
